# Supplementary material for: G6PD deficiency in Malaysia’s Proto-Malay Orang Asli indigenous population: A molecular and epidemiological study
Source: PLoS One. 2025 Oct 10;20(10):e0334185. doi: 10.1371/journal.pone.0334185 (PMC12513647; doi:10.1371/journal.pone.0334185)
Supplement: S1 File — The study’s minimal underlying G6PD activity data set. (PDF) [file pone.0334185.s001.pdf]

Number	G6PD activity by OSMMR2000-D (IU/gHb)
1	1.3
2	7.0
3	9.7
4	3.8
5	9.4
6	8.6
7	8.2
8	11.6
9	9.4
10	11.9
11	9.6
12	10.2
13	8.6
14	9.8
15	10.3
16	9.1
17	9.9
18	9.0
19	8.8
20	4.7
21	8.8
22	8.8
23	8.4
24	3.7
25	8.8
26	20.8
27	9.2
28	8.9
29	9.9
30	9.1
31	7.7
32	10.3
33	2.0

34	10.4
35	9.3
36	9.7
37	9.9
38	8.1
39	10.4
40	10.5
41	7.5
42	8.7
43	1.1
44	8.0
45	12.2
46	8.5
47	8.8
48	12.1
49	9.0
50	8.8
51	7.8
52	13.7
53	10.6
54	10.4
55	7.8
56	8.5
57	8.3
58	9.1
59	9.8
60	9.8
61	10.3
62	9.7
63	8.5
64	8.6
65	9.2
66	7.6

67	10.3
68	10.2
69	1.1
70	9.9
71	11.1
72	15.7
73	9.3
74	10
75	10.6
76	2.9
77	11.5
78	9.5
79	0.1
80	9.2
81	8.9
82	13.2
83	11.4
84	9.6
85	11.3
86	10.7
87	9.5
88	9.3
89	8.8
90	9.7
91	9.7
92	2.4
93	10.2
94	12.7
95	1.7
96	7.8
97	8.6
98	0.9
99	2.1
100	6.2
101	13.4

102	8.4
103	11.6
104	10.4
105	9.1
106	10.5
107	9.8
108	2.7
109	8
110	10.6
111	9.2
112	2.5
113	9.2
114	8.6
115	10.7
116	8.9
117	9.5
118	9.2
119	9.3
120	9.4
121	9.2
122	9.4
123	10
124	10.4
125	9.5
126	10.8
127	9.5
128	7.9
129	1.1
130	11.7
131	10.4
132	9.9
133	9.9
134	10.9
135	7.3
136	10

137	10.6
138	11
139	11.7
140	8.3
141	12.7
142	10.8
143	12
144	9.8
145	9.8
146	10.9
147	9.2
148	7.2
149	15.1
150	10.2
151	10.6
152	10.4
153	9.2
154	13.8
155	9.4
156	9.8
157	5
158	9.4
159	8.7
160	13.8
161	11.8
162	11.7
163	10.7
164	13.1
165	11.8
166	11.1
167	12
168	13.9
169	11.8
170	6.8
171	10.7

172	12.6
173	11.8
174	13.2
175	11.4
176	12.8
177	12.7
178	10.4
179	11.8
180	10.1
181	12.1
182	13.1
183	11.2
184	11.1
185	7.7
186	10.7
187	5.6
188	14.2
189	14
190	8
191	7.1
192	10.1
193	14.5
194	11.2
195	12.4
196	12.2
197	12.1
198	10.1
199	11.7
200	12.6
201	11.9
202	13.9
203	11.7
204	10.1
205	10.8
206	10.3

207	12.2
208	3.6
209	1.4
210	8.6
211	10.7
212	11.5
213	11.3
214	9.6
215	11.4
216	8.6
217	3.2
218	9.3
219	9.7
220	9.5
221	9.5
222	9.4
223	11.0
224	3.1
225	10.9
226	11.2
227	11.0
228	11.4
229	13.9
230	9.1
231	9.1
232	10.3
233	11.0
234	9.7
235	10.0
236	6.9
237	10
238	9.5
239	1.5
240	12.9
241	4.2

242	12.4
243	11.8
244	1.4
245	13.4
246	12.4
247	12.1
248	13.8
249	13.8
250	10.7
251	11.9
252	11.7
253	12.2
254	10.9
255	1.5
256	11.5
257	11.3
258	11
