# Supplementary material for: G6PD deficiency in Malaysia’s Proto-Malay Orang Asli indigenous population: A molecular and epidemiological study
Source: PLoS One. 2025 Oct 10;20(10):e0334185. doi: 10.1371/journal.pone.0334185 (PMC12513647; doi:10.1371/journal.pone.0334185)
Supplement: S2 File — The study’s minimal underlying genetic dataset, presented as a consolidated table of all detected mutations with corresponding allele frequencies, including variants in intronic and untranslated regions. (PDF) [file pone.0334185.s002.pdf]

S1 File. Detected mutations and allelic frequencies in the Malaysian Proto Malay Orang Asli population. The study's minimal underlying genetic dataset, presented as a consolidated table of all detected mutations with corresponding allele frequencies, including variants in intronic and untranslated regions.

Type	G6PD Mutation / Variant	dbSNP ID	Genomic Location (GRCh38)	Male (N)	Heterozygous Female (N)	Homozygous Female (N)	Compound Heterozygous Female (N)	Total Mutant Allele (N)	Allelic Frequency (%)	Functional Classification
Exonic (missense)	G6PD Viangchan (871G>A)	rs137852327	chrX:154535780	7	5	2	0	16	36.4	Pathogenic
	G6PD Coimbra (592C>T)	rs137852328	chrX:154536059	4	7	1	1	14	31.8	Pathogenic
	G6PD Union (1360C>T)	rs766421313	chrX:154534981	5	6	0	0	11	25.0	Pathogenic
	G6PD Kaiping (1388G>A)	rs72554665	chrX:154534953	1	0	0	1	2	4.6	Pathogenic
	rs782038151	rs782038151	chrX:154535364	1	0	0	0	1	2.3	Uncertain significance
Intronic	Variant ID: 982298	rs982298	chrX:154532293	12	16	0	0	28	70.0	Benign
	Variant ID: 1780243	rs1780243	chrX:154538857	0	1	0	0	1	2.5	Benign

	Unknown intronic variants	—	—	3	5	0	0	8	20.0	Unknown significance
3′UTR	Variant ID: 1780209 (c.1792C>G)	rs1780209	chrX: 94991941	0	5	0	0	5	12.5	Uncertain significance
	3′UTR variants (VUS)	—	—	0	30	0	0	30	75.0	Uncertain significance
